# 基于老年营养风险指数构建老年非小细胞肺癌患者的预后模型

**DOI:** 10.3779/j.issn.1009-3419.2023.106.14

**Published:** 2023-07-20

**Authors:** Xiaonan ZHANG, Yajun XIONG, Aiguo XU

**Affiliations:** 450052 郑州，郑州大学第一附属医院呼吸与危重症医学科; Department of Respiratory and Critical Care Medicine, the First Affiliated Hospital of Zhengzhou University, Zhengzhou 450052, China

**Keywords:** 老年营养风险指数, 老年, 肺肿瘤, 总体生存率, 营养状况, Geriatric nutritional risk index, Elderly, Lung neoplasms, Overall survival rate, Nutritional status

## Abstract

**背景与目的** 癌症患者的营养状况与预后之间的关系已成为研究关注的热点。本研究旨在探索老年营养风险指数（geriatric nutritional risk index, GNRI）在老年非小细胞肺癌（non-small cell lung cancer, NSCLC）患者预后评估中的应用价值，构建预测老年NSCLC患者预后的列线图模型。**方法** 回顾性分析郑州大学第一附属医院于2016年1月至2019年12月初治的、≥65岁的153例NSCLC患者的资料。使用受试者工作特征（receiver operating characteristic, ROC）曲线确定GNRI的最佳截断值，把患者分为高GNRI组和低GNRI组，通过Kaplan-Meier曲线和Log-rank检验对两组患者总生存期（overall survival, OS）进行比较。使用单因素和多因素Cox回归分析探讨老年NSCLC患者不良预后的危险因素，利用R软件构建预测老年NSCLC患者生存率的列线图模型，并验证该模型。**结果** 高GNRI组和低GNRI组在年龄、性别、体重指数（body mass index, BMI）、组织学类型、白蛋白水平、治疗方式、中性粒细胞与淋巴细胞比值（neutrophil to lymphocyte ratio, NLR）、预后营养指数（prognostic nutritional index, PNI）、全身免疫炎症指数（systemic immune-inflammation index, SII）和细胞角蛋白19片段（cytokeratin 19 fragment, CYFRA21-1）方面存在统计学差异（P<0.05）。Kaplan-Meier曲线显示低GNRI组的患者总生存期较短。多因素Cox回归分析显示CYFRA21-1>3.3 ng/mL是影响NSCLC患者OS的独立危险因素，GNRI>97.09是保护因素[风险比（hazard ratio, HR）=0.52，95%置信区间（confidence interval, CI）：0.34-0.79，P<0.05]。IV期患者发生死亡的风险是I期患者的1.98倍（95%CI: 1.02-3.86, P<0.05）。与行综合治疗的患者相比，行单一化疗的患者发生死亡的风险增加3.58倍（95%CI: 2.03-6.32, P<0.05）。基于GNRI构建的列线图模型对老年NSCLC患者OS进行预测，其一致性指数（concordance index, C-index）为0.70（95%CI: 0.65-0.76），预测1、2年生存率的曲线下面积（area under the curve, AUC）分别为0.93（95%CI: 0.87-0.98）、0.72（95%CI: 0.63-0.80），校准曲线显示该模型的预测符合度较好。**结论** 高GNRI评分与改善老年NSCLC患者的生存率显著相关，依靠截断值可能提供恰当的营养支持时机。该研究构建的列线图可作为一种有效预测老年NSCLC患者生存率的工具，具有很强的临床实用性。

## Abstract (en)

**Background and objective** The relationship between nutritional status and prognosis of cancer patients has emerged as a hotspot for research. The aim of this study is to explore the application value of the geriatric nutritional risk index (GNRI) in assessing the prognosis of elderly patients with non-small cell lung cancer (NSCLC), and establish a Nomogram to predict the prognosis of elderly patients with NSCLC. **Methods** The data of patients with NSCLC aged ≥65 years who were initially treated in the First Affiliated Hospital of Zhengzhou University from January 2016 to December 2019 were retrospectively analyzed. To determine the optimal cut-off value for GNRI, receiver operating characteristic (ROC) curve was constructed, and the patients were divided into high and low GNRI groups. Kaplan-Meier curve and Log-rank test were used to compare overall survival (OS) of the two groups. Univariate and multivariate Cox regression was used to analyze the risk factors for poor prognosis in elderly patients with NSCLC. Nomogram predicting survival in elderly patients with NSCLC was constructed and validated by using R software. **Results** Statistically significant differences in age, gender, body mass index (BMI), histological type, albumin, treatment methods, neutrophil to lymphocyte ratio (NLR), prognostic nutritional index (PNI), systemic immune-inflammation index (SII) and cytokeratin 19 fragment (CYFRA21-1) were observed between the high and low GNRI groups (P<0.05). The Kaplan-Meier curve showed a shorter OS in the low-GNRI group. Multivariate Cox regression analysis showed that CYFRA21-1>3.3 ng/mL was an independent risk factor for the development of OS in patients with NSCLC, and GNRI>97.09 was a protective factor [hazard ratio (HR)=0.52, 95% confidence interval (CI): 0.34-0.79, P<0.05]. Patients in the stage IV had a 1.98-fold increased risk of death compared with patients in the stage I (95%CI: 1.02-3.86, P<0.05). The risk of death was 3.58 times higher in patients receiving chemotherapy alone compared with those receiving combination therapy (95%CI: 2.03-6.32, P<0.05). A Nomogram constructed on the basis of GNRI, which predicted the OS of elderly patients with NSCLC with a concordance index (C-index) of 0.70 (95%CI: 0.65-0.76), and the area under the curve (AUC) for 1 and 2-year survival rates to be 0.93 (95%CI: 0.87-0.98) and 0.72 (95%CI: 0.63-0.80), respectively, and the calibration curve has a good coincidence of prediction. **Conclusion** High GNRI scores are significantly associated with improved survival in elderly patients with NSCLC, and reliance on cut-off values may provide the appropriate timing for nutritional support. The Nomogram constructed in this study can be used as an effective tool to predict the survival rate of elderly patients with NSCLC, which has strong clinical practicability.

全球^[1]^和中国^[2]^癌症统计数据均表明肺癌仍是最常见的癌症之一，也是造成癌症患者相关死亡的主要原因。近年来，癌症患者的营养状况与预后之间的关系已成为研究热点。肺癌患者营养不良发生率为30%-60%^[3⇓-5]^，这取决于不同的评估方法。老年患者（≥65岁）更易发生营养不良^[6]^，由于其整体机能衰退，与年轻患者相比，老年患者营养状况恶化更快，甚至出现恶病质。Dunne等^[7]^表明65%的老年癌症患者存在恶病质，与更差的预后相关。因此，准确评估营养状况并识别高营养风险患者可能会改善老年非小细胞肺癌（non-small cell lung cancer, NSCLC）患者的预后。

目前营养评估大多取决于对患者的主观评估或需要计算大量参数^[8]^。因此，建立一个简便有效的营养评估指标是必要的。Bouillanne等^[9]^提出的老年营养风险指数（geriatric nutritional risk index, GNRI）是一种评估老年住院患者发病率和死亡率的指标，由血清白蛋白、身高和体重决定，其用于评估恶性或非恶性疾病患者的短期或长期预后，包括心脏^[10]^、肾脏^[11]^、呼吸系统^[12]^疾病及某些癌症（结直肠癌^[13]^、食管癌^[14]^、胰腺癌^[15]^等）。有研究^[16]^表明术前GNRI可作为识别肺癌术后复发、术后并发症及预后的指标。然而，GNRI对于老年NSCLC患者的意义尚不完全明确。因此本研究旨在探索GNRI在老年NSCLC患者预后评估中的应用价值，并构建一个预测老年NSCLC患者预后的列线图模型。

## 1 资料与方法

### 1.1 研究对象

本研究对2016年1月至2019年12月于郑州大学第一附属医院确诊的153例NSCLC患者资料进行回顾性分析。纳入标准：（1）初次诊断的NSCLC患者，并通过病理证实；（2）年龄：65岁及以上；（3）治疗前临床资料完整；（4）可随访到总生存期（overall survival, OS）。排除标准：（1）合并其他系统肿瘤；（2）合并自身免疫性疾病；（3）合并活动性感染；（4）失访。本研究已通过郑州大学第一附属医院伦理委员会的批准（批准号：2023-KY-0359-001）。

### 1.2 数据收集

通过医院电子病历系统收集患者确诊时的年龄、身高、体重、吸烟史、肿瘤原发灶-淋巴结-转移（tumor-node-metastasis, TNM）分期、肿瘤的组织学类型、治疗方式、治疗前的血清白蛋白、血常规、肿瘤标志物等。肺癌分期根据2015年国际肺癌研究协会（International Association for the Study of Lung Cancer, IASLC）制定的第八版肺癌TNM分期标准。

### 1.3 定义

GNRI=1.489×白蛋白（g/L）+41.7×实际体重/理想体重。理想体重由Lorenz方程计算，对于男性：身高-100-[（身高-150）/4]；对于女性：身高-100-[（身高-150）/2.5]。当实际体重超过理想体重时，实际体重/理想体重设定为1。体重指数（body mass index, BMI）=体重（kg）/身高^2^（m）。过轻：BMI<18.5 kg/m^2^；正常：18.5 kg/m^2^≤BMI<24.0 kg/m^2^；超重：24.0 kg/m^2^≤BMI<28.0 kg/m^2^；肥胖：BMI≥28.0 kg/m^2^。预后营养指数（prognostic nutritional index, PNI）=血清白蛋白（g/L）+5×外周血淋巴细胞总数（×10^9^/L）。全身免疫炎症指数（systemic immune-inflammation index, SII）=血小板计数×中性粒细胞计数/淋巴细胞计数（×10^9^/L）。中性粒细胞与淋巴细胞比值（neutrophil to lymphocyte ratio, NLR）=中性粒细胞计数/淋巴细胞计数。血小板与淋巴细胞比值（platelet to lymphocyte ratio, PLR）=血小板计数/淋巴细胞计数。

### 1.4 随访

对所有入组患者进行随访，OS为本研究的主要结局。OS定义为从确诊当天至死亡或末次随访时间。每3个月评价一次生存结局，末次随访时间为2022年10月30日。信息来源包括医院住院或门诊记录、患者或家属电话、短信联系等。

### 1.5 统计方法

利用MedCalc 19.0.4绘制受试者工作特征（receiver operating characteristic, ROC）曲线确定GNRI、NLR、PLR、PNI、SII的最佳截断值。数据分析使用SPSS 26.0和R 4.1.2进行处理，呈正态分布的计量资料以均数±标准差表示，并采用独立样本t检验或单因素方差分析进行组间比较；呈偏态分布的计量资料以中位数和四分位数间距M（Q1, Q3）表示，组间比较采用Mann-Whitney U检验。计数资料以例数（百分比）表示，并使用χ^2^或Fisher确切检验进行组间比较。通过Kaplan-Meier方法和Log-rank检验比较OS，通过单因素和多因素Cox回归分析识别与OS相关的变量。利用筛选出的独立危险因素建立预测老年NSCLC患者生存率的列线图模型，使用Bootstrap重复自抽样法进行内部验证。根据校准曲线评估模型的校准能力，时间依赖性ROC（time-dependent ROC, tdROC）曲线评估模型的预估准确度，应用决策曲线分析（decision curve analysis, DCA）对净收益进行评估。统计学分析均采用双侧检验，P<0.05被认为差异有统计学意义。

## 2 结果

### 2.1 临床特征

153例患者中，男性99例（64.7%），女性54例（35.3%），中位年龄为69岁。83例患者（54.2%）的体重指数在正常范围内，14例（9.2%）低于正常水平，46例（30.1%）处于超重水平，10例（6.5%）处于肥胖水平。I、II、III、IV期的患者分别为29例（19.0%）、19例（12.4%）、33例（21.6%）、72例（47.0%）。最常见的组织学类型为腺癌，发生率为62.1%（95例患者）。截止到随访日期，死亡患者101例（66.0%），中位生存期为30个月。1、2、3年生存率分别为93.5%、60.8%和41.8%。

### 2.2 确定最佳截断值

利用MedCalc软件绘制ROC曲线确定GNRI、NLR、PLR、PNI、SII的最佳截断值（图1）。ROC曲线显示GNRI的最佳截断值为97.09[曲线下面积（area under the curve, AUC）=0.74，95%置信区间（confidence interval, CI）：0.66-0.81，P<0.001]。NLR的最佳截断值为4.11（AUC=0.63, 95%CI: 0.55-0.71, P<0.05）。PLR的最佳截断值为161.468（AUC=0.62, 95%CI: 0.54-0.70, P<0.05）。SII的最佳截断值为694.602（AUC=0.64, 95%CI: 0.56-0.72, P<0.05）。PNI的最佳截断值为44.45（AUC=0.71, 95%CI: 0.63-0.78, P<0.001）。

**图1:**
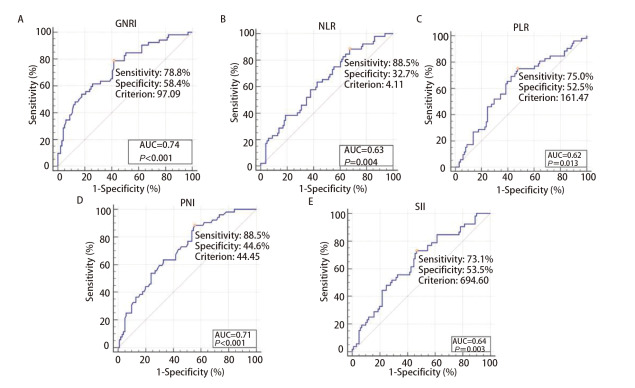
MedCalc软件确定GNRI（A）、NLR（B）、PLR（C）、PNI（D）、SII（E）的最佳截断值

### 2.3 GNRI与患者基线特征之间的关系

根据MedCalc软件确定的GNRI最佳截断值，将153例患者分为两组，分别为高GNRI组（GNRI>97.09）、低GNRI组（GNRI≤97.09）。两组患者在年龄、性别、BMI、组织学类型、白蛋白水平、治疗方式、NLR、SII、PNI和细胞角蛋白19片段（cytokeratin 19 fragment, CYFRA21-1）上有差异，且差异具有统计学意义（P<0.05），而在吸烟状况、TNM、癌胚抗原（carcinoembryonic antigen, CEA）、PLR方面无统计学差异（P>0.05）（表1）。

**表1:** 两组NSCLC患者基线资料比较

Variable	Category	Total (n=153)	GNRI			Z/χ^2^	P
			>97.09 (High) (n=83)	≤97.09 (Low) (n=70)			
Age (yr)		69 ( 67, 73)	68 (66, 72)	70 (67, 75)		2.03^#^	0.04
Gender	Male	99 (64.7%)	44 (53.0%)	55 (78.6%)		10.86^*^	0.01
	Female	54 (35.3%)	39 (47.0%)	15 (21.4%)			
Smoking	Never	74 (48.4%)	46 (55.4%)	28 (40.0%)		3.62^*^	0.06
	Ever or current	79 (51.6%)	37 (44.6%)	42 (60.0%)			
BMI (kg/m^2^)	Underweight (<18.5)	14 (9.2%)	2 (2.4%)	12 (17.1%)		14.80^*^	0.01
	Normal (18.5 to <24.0)	83 (54.2%)	42 (50.6%)	41 (58.6%)			
	Overweight (24.0 to <28.0)	46 (30.1%)	32 (38.6%)	14 (20.0%)			
	Obese (≥28.0)	10 (6.5%)	7 (8.4%)	3 (4.3%)			
Histology	AC	95 (62.1%)	60 (72.3%)	35 (50.0%)		8.05^*^	0.02
	SCC	51 (33.3%)	20 (24.1%)	31 (44.3%)			
	Others	7 (4.6%)	3 (3.6%)	4 (5.7%)			
TNM stage	I	29 (19.0%)	18 (21.7%)	11 (15.7%)		4.93^*^	0.18
	II	19 (12.4%)	7 (8.4%)	12 (17.2%)			
	III	33 (21.6%)	15 (18.1%)	18 (25.7%)			
	IV	72 (47.0%)	43 (51.8%)	29 (41.4%)			
Treatment	Single surgery	7 (4.6%)	6 (7.2%)	1 (1.4%)		8.92^*^	0.03
	Single chemotherapy	18 (11.8%)	5 (6.0%)	13 (18.6%)			
	Single targeted therapy	16 (10.5%)	11 (13.3%)	5 (6.0%)			
	Combined therapy	112 (73.2%)	61 (73.5%)	51 (72.9%)			
Albumin (g/L)	≤35	24 (15.7%)	0 (0.0%)	24 (34.3%)		33.75^*^	<0.001
	>35	129 (84.3%)	83 (100.0%)	46 (65.7%)			
NLR	≤4.11	114 (74.5%)	70 (84.3%)	44 (62.9%)		9.23^*^	0.01
	>4.11	39 (25.5%)	13 (15.7%)	26 (37.1%)			
PLR	≤161.47	87 (56.9%)	53 (63.9%)	34 (48.6%)		3.62^*^	0.06
	>161.47	66 (43.1%)	30 (36.1%)	36 (51.4%)			
SII	≤694.60	85 (55.6%)	55 (66.3%)	30 (42.9%)		8.43^*^	0.01
	>694.60	68 (44.4%)	28 (33.7%)	40 (57.1%)			
PNI	≤44.45	51 (33.3%)	7 (8.4%)	44 (62.9%)		50.61^*^	<0.001
	>44.45	102 (66.7%)	76 (91.6%)	26 (37.1%)			
CYFRA21-1 (ng/mL)	≤3.3	59 (38.6%)	45 (54.2%)	14 (20.0%)		18.77^*^	<0.001
	>3.3	94 (61.4%)	38 (45.8%)	56 (80.0%)			
CEA (ng/mL)	≤5	81 (52.9%)	41 (49.4%)	40 (57.1%)		0.91^*^	0.34
	>5	72 (47.1%)	42 (50.6%)	30 (42.9%)			

^#^: statistical magnitude Z; *: statistical magnitude χ^2^; NSCLC: non-small cell lung cancer;BMI: body mass index; AC: adenocarcinoma; SCC: squamous cell carcinoma; CYFRA21-1: cytokeratin 19 fragment; CEA: carcinoembryonic antigen.

### 2.4 GNRI与OS之间的关联

在Kaplan-Meier曲线中，生存曲线按GNRI分组显著分层，GNRI低与OS差显著相关。高GNRI组的中位生存时间（46个月）是低GNRI组（21个月）的2.2倍，高GNRI组1、2、3年生存率分别为98.8%、75.9%、54.2%，低GNRI组1、2、3年生存率分别为85.7%、42.9%、27.0%，两组差异有统计学意义（P<0.0001）（图2A）。

**图2:**
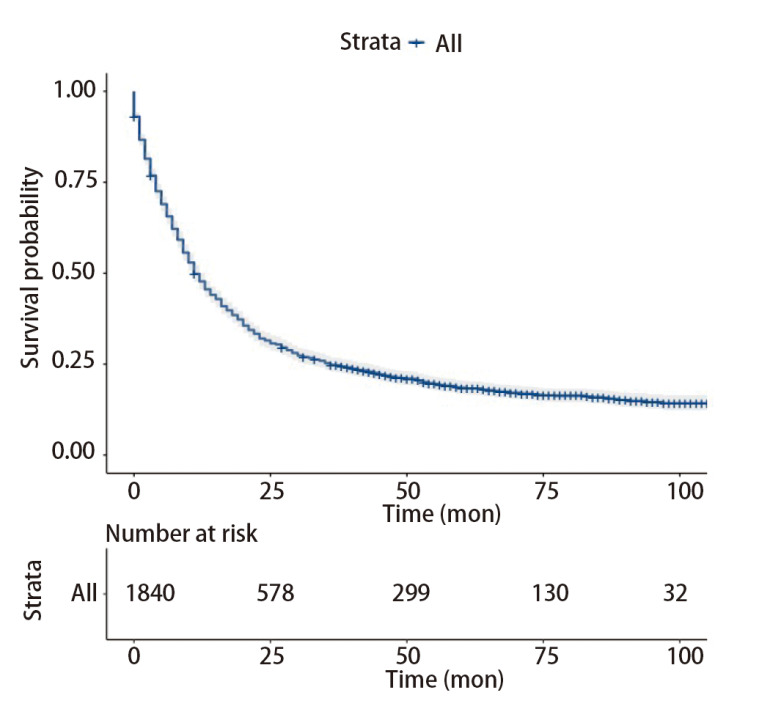
总人群中两组老年NSCLC患者的生存曲线（A）和基于有无转移的老年NSCLC患者的生存曲线，包括未发生转移组（B）和发生转移组（C）。

### 2.5 影响总生存率的单因素和多因素分析

单因素Cox回归分析（表2）显示TNM分期、血清白蛋白、GNRI、NLR、PLR、SII、PNI、CYFRA21-1与OS相关，其中GNRI>97.09、PNI>44.45、血清白蛋白>35 g/L是保护性因素。将单因素分析中具有显著差异的相关指标进行相关性分析，发现血清白蛋白（r=0.93, P<0.001）和PNI（r=0.75, P<0.001）和BMI（r=0.54, P<0.001）与GNRI存在共线性，故不纳入血清白蛋白、PNI、BMI进入多因素Cox回归分析中。相关分析显示NLR（r=0.82, P<0.001）和PLR（r=0.65, P<0.001）与SII存在共线性，故不纳入NLR、PLR进入多因素Cox回归分析中。多因素Cox回归分析（表2）显示CYFRA21-1>3.3 ng/mL是影响NSCLC患者总体生存率的独立危险因素，GNRI>97.09是保护因素，GNRI越高，死亡的风险越小[风险比（hazard ratio, HR）=0.52，95%CI：0.34-0.79，P<0.05]。与I期患者相比，IV期患者发生死亡的风险增加1.98倍（95%CI: 1.02-3.86, P<0.05）。与接受综合治疗的患者相比，接受单一化疗的患者死亡发生风险增加3.58倍（95%CI: 2.03-6.32, P<0.05）。

**表2:** 老年NSCLC患者OS的单因素、多因素Cox回归分析

Variable	Univariate analysis			Multivariate analysis	
	HR (95%CI)	P		HR (95%CI)	P
Age	1.03 (0.98-1.08)	0.21			
Gender (Female vsmale)	0.74 (0.49-1.13)	0.16			
Smoking (Ever or current vs never)	1.18 (0.80-1.75)	0.39			
GNRI (High vs low)	0.38 (0.26-0.57)	<0.001		0.52 (0.34-0.79)	<0.05
BMI (vs normal)					
Underweight	2.25 (1.20-4.21)	0.01			
Overweight	0.63 (0.39-1.01)	0.05			
Obese	0.67 (0.27-1.66)	0.38			
Stage (vs I)					
II	2.41 (1.11-5.21)	0.03		1.49 (0.67-3.29)	0.33
III	2.26 (1.12-4.56)	0.02		1.81 (0.87-3.75)	0.11
IV	2.56 (1.36-4.80)	<0.05		1.98 (1.02-3.86)	0.04
Histology (vs AC)					
SCC	1.31 (0.87-1.99)	0.20			
Others	1.07 (0.39-2.97)	0.89			
Treatment (vscombined therapy)					
Surgery	0.14 (0.02-0.99)	0.04		0.34 (0.05-2.58)	0.30
Chemotherapy	3.69 (2.15-6.34)	<0.001		3.58 (2.03-6.32)	<0.001
Targeted therapy	0.89 (0.47-1.68)	0.71		1.08 (0.56-2.09)	0.81
Albumin (>35 g/L vs≤35 g/L)	0.41 (0.25-0.67)	<0.001			
NLR (>4.11 vs ≤4.11 )	1.95 (1.29-2.96)	<0.05			
PLR (>161.47 vs≤161.47)	2.31 (1.56-3.43)	<0.001			
SII (>694.6 vs ≤694.60)	1.96 (1.33-2.90)	<0.05		1.38 (0.91-2.10)	0.13
PNI (>44.45 vs ≤44.45)	0.46 (0.31-0.68)	<0.001			
CYFRA21-1 (>3.3 ng/mL vs ≤3.3 ng/mL)	2.51 (1.62-3.90)	<0.001		1.81 (1.13-2.89)	0.01
CEA (>5 ng/mL vs ≤5 ng/mL)	1.47 (0.99-2.19)	0.06			

HR: hazard ratio; CI: confidence interval.

大部分肺癌患者在诊断时已经发生转移，发生转移与不良预后显著相关。根据有无发生转移，我们进一步对GNRI进行亚组分析，结果显示肿瘤无论是否发生转移，高GNRI组都有着更好的预后（P<0.0001）（图2B，图2C）。

### 2.6 列线图模型的建立及评价

根据多因素Cox回归分析得出的结果，建立了包括GNRI、TNM分期和CYFRA21-1治疗方式在内的列线图（图3A）。基于列线图预测总分，将患者分为低、中、高风险三组（低风险组：20-75；中风险组：75-120；高风险组：120-max），并绘制了生存曲线，结果表明低风险组比中、高风险组的预后好（P<0.001）（图3B）。经过Bootstrap重复自抽样法进行内部验证，表明该列线图模型的一致性指数（concordance index, C-index）为0.70（95%CI: 0.65-0.76）。预测1、2年生存率的校准曲线显示出了良好的预测符合度（图4A）。该模型预测1、2年生存率的AUC分别为0.93（95%CI: 0.87-0.98）、0.72（95%CI: 0.63-0.80）（图4B）。决策曲线分析表示，与TNM分期、治疗方式、CYFRA21-1相比，该模型及GNRI在预测患者1、2年总体生存率方面具有更好的性能（图4C）。

**图3:**
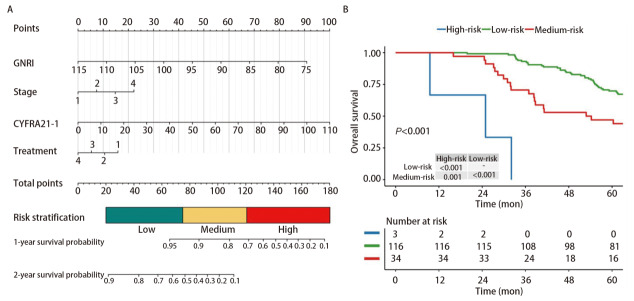
列线图及危险分层。A：老年NSCLC患者的预后列线图（治疗方式赋值：1=单纯手术，2=单纯化疗，3=单纯靶向，4=综合治疗；分期赋值：1=I期，2=II期，3=III期；4=IV期）；B：根据列线图总分分层后不同风险组的生存曲线。

**图4:**
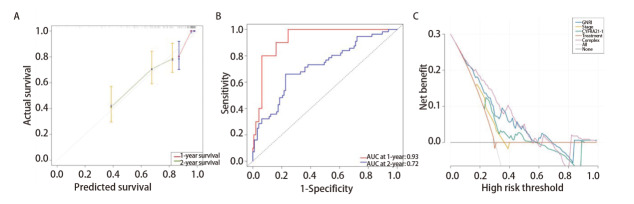
列线图性能评价。A：预后列线图预测总体生存率的校准曲线；B：列线图预测总体生存率的tdROC；C：决策曲线分析模型的临床应用价值。

## 3 讨论

考虑到NSCLC的巨大负担和不良预后，迫切需要识别和验证这些患者特异性的预后因素。近年来，研究人员越来越关注治疗前营养不良在癌症患者存活中的作用。有研究^[17]^表明营养状况与癌症患者生存率相关。本研究旨在确定治疗前GNRI水平在预测老年NSCLC患者OS中的价值。我们的研究证明了低GNRI与较差的OS相关。建立治疗前的营养评估对预测老年NSCLC患者的预后至关重要，同时也可能为营养支持提供恰当时机。

GNRI是由Bouillanne等^[9]^提出的一种新的营养评估工具。体重是GNRI的一个组成部分。肿瘤患者出现体重减轻是因为在静息状态下他们的能量消耗明显高于普通人群，研究^[18]^表明体重持续减轻超过2.4%就会显著影响肿瘤患者的生存期。体重减轻是最早用于肺癌患者营养评价的指标之一，Trestini等^[19]^研究表明基线体重减轻对NSCLC患者的OS产生不利影响。但是由于该信息易受回忆偏差的影响，容易导致结果出现偏差。我们的研究只需测量患者治疗前的一次体重、身高，数据易获取且真实可靠。

BMI也是反映机体体重、营养状况的指标，甚至比GNRI更易获取。一项荟萃分析^[20]^提供了BMI增加是肺癌患者保护因素的定量证据。由于“肥胖悖论”的存在，BMI对癌症患者预后的影响存在争议。Dahlberg等^[21]^发现了一种时间依赖性关系，随访早期肥胖导致接受化疗的IV期NSCLC患者结局改善，但16个月后死亡风险增加。Shepshelovich等^[22]^的研究表明在对肺癌患者的评估中，诊断时体重过轻或病态肥胖均与较低的生存率相关。我们的研究发现BMI过低是影响肺癌患者生存的危险因素（HR=2.25, 95%CI: 1.20-4.21, P<0.05），这与上述相关研究结论一致；超重和肥胖与肺癌患者的生存率无相关性。这些都表明了单独使用BMI作为NSCLC预后指标的复杂性。

血清白蛋白是GNRI的另一个组成部分，且加权值比体重更大。血清白蛋白作为一种表示长期营养状态的生化标志物，在癌症患者中提供有用的预后意义，可用于临床更好地定义癌症患者的基线风险^[23]^。血清白蛋白还具有免疫调节、抗氧化^[24,25]^和潜在的抗肿瘤作用^[26]^。一些研究^[27,28]^表明较高的血清白蛋白水平与较好的生存率相关。我们的研究表明血清白蛋白与OS在单因素Cox回归分析中相关，因共线性问题未将此纳入多因素Cox回归分析中。因此，我们认为与单独使用BMI或血清白蛋白相比，GNRI可能是一个更可靠的预后因素。

GNRI被认为是多维的，可以反映机体的营养状态、免疫状况和炎症状况。Karayama等^[29]^通过前瞻性研究证实GNRI与接受纳武利尤单抗治疗的晚期NSCLC患者的OS和无进展生存期（progression-free survival, PFS）有关，营养状况的评估可能有助于预测免疫检查点抑制剂（immune checkpoint inhibitors, ICIs）的疗效。Shoji等^[30]^通过多中心回顾性研究表明术前GNRI是一种新的术后合并症的预测因子，也是一种可识别高风险老年NSCLC患者的预后因素。有研究^[31,32]^表明术前GNRI水平有助于预测肺癌术后生存率。一项研究^[33]^报告了GNRI是晚期NSCLC患者一个重要的预后因素，且与年龄无关。我们的研究涵盖了所有分期的患者，无论接受何种治疗。一项回顾性研究^[34]^也报告了GNRI在预测老年转移性肺腺癌患者OS中的重要性，我们的研究根据亚组分析可见未发生转移组GNRI水平与OS也显著相关，发生转移组不仅包括腺癌，还包括鳞癌、大细胞癌等。这些研究都支持我们的发现，即GNRI是肺癌患者较好的预后生物标志物。

多因素Cox回归显示GNRI、TNM分期和CYFRA21-1、治疗方式是老年NSCLC患者的独立预后因素。基于这几个独立危险因素，我们建立了相应的列线图模型。列线图是基于多个预后因素构建的，比单个因素效果更好。该模型在老年NSCLC患者OS预测中表现良好，因此，我们相信它有潜力帮助医生做出医疗决策，可以帮助医生更准确地评估患者预后。

CYFRA21-1是细胞角蛋白19的可溶性片段，作为NSCLC最有价值的血清肿瘤标志物，因其易于检测而被常规应用于肺癌的诊断和预后。有研究^[35]^表明基线时高水平的CYFRA21-1与晚期NSCLC患者的生存率较差相关。与其他肿瘤标志物如CEA、神经元特异性烯醇化酶（neuron specific enolase, NSE）等联合检测可以更准确预测肺癌的诊断和预后^[36]^。

我们的研究中多种反映系统炎症的指标，如NLR、PLR、SII等，在以GNRI不同分组中均具有统计学差异，这揭示了营养不良状态可能与炎症反应、免疫反应等有关，可能是营养不良导致不良预后的原因之一，这需要深入的研究进行验证。该研究存在如下几个局限性。第一，这是一项回顾性研究，来自单一机构，样本量相对较小，存在一定的筛选和信息偏差，因此需要进行多中心前瞻性研究和更大样本的临床分析，包括营养干预，以确保这些结果的准确性；第二，本研究仅包括≥65岁的老年NSCLC患者，结果并不代表非老年和其他类型的肺癌患者；第三，本研究以ROC曲线获得GNRI的截断值特异度低，为58.4%，需要增加样本量或联合其他指标进一步提高特异度；第四，治疗信息只收录了治疗方式，无具体的方案，这些也可能会影响患者的生存预后；第五，由于随访至2年时样本量较大，本研究列线图只预测了1、2年生存率，且本研究对列线图模型只进行了内部验证，还需进行多中心验证和外部验证，以进一步提高模型预测的准确性。因此，我们需要前瞻性研究，使用大型队列，对老年NSCLC患者进行更长时间的随访和标准化治疗，以验证我们的发现。

综上，我们的研究表明较高的GNRI评分与改善老年NSCLC患者的生存率显著相关，依靠截断值可能为营养支持提供恰当的时机。本研究构建的列线图可作为一种有效预测老年NSCLC患者生存率的模型，具有很强的临床实用性。

**Competing interests**

The authors declare that they have no competing interests.

**Author contributions**

Zhang XN and Xu AG conceived and designed the study. Zhang XN and Xiong YJ collected clinical data and made follow-up. Zhang XN and Xiong YJ analyzed the data. Zhang XN wrote the manuscript. All authors had access to the data. All authors read and approved the final manuscript as submitted.
